# Case Report: Autistic child with restrictive eating behaviour, limping gait and erythematous gingival mass-scurvy?

**DOI:** 10.3389/frcha.2025.1600861

**Published:** 2025-07-18

**Authors:** Preeyanikaa Logonathan, Nurhidayah Muhd Noor, Aminah Marsom

**Affiliations:** Department of Paediatric Dentistry, Selayang Hospital (Ministry of Health), Batu Caves, Selangor, Malaysia

**Keywords:** autism, eating behaviour, limping, gingival swelling, scurvy

## Abstract

Scurvy, a condition caused by a deficiency in ascorbic acid, is often considered an outdated, textbook disease, largely forgotten in the 21st century. However, recent reports indicate a rise in cases, particularly among individuals with risk factors for nutritional deficiencies or those with specific dietary habits. These cases are frequently misdiagnosed, leading to a series of unnecessary tests that could be avoided with a thorough assessment of dietary intake. In this report, we present the case of a 10-year-old with autism spectrum disorder (ASD) and a selective eating pattern, who presented with a limping gait, tenderness in the right calf, and significant weight loss. A skin examination revealed multiple perifollicular hemorrhages with corkscrew-shaped hair, as well as localized erythematous and hypertrophic gingiva in all four quadrants. Based on clinical findings, scurvy was suspected, and vitamin C supplementation was initiated both for diagnostic and therapeutic purposes. The diagnosis was confirmed when serum vitamin C levels were found to be critically low (5 µmol/L, normal range: 28–120 µmol/L). The patient's response to vitamin C was impressive, with complete gingival healing and noticeable weight gain within three weeks. Although scurvy is often thought of as an ancient disease, it has seen a resurgence, posing diagnostic challenges due to its diverse clinical manifestations. Early diagnosis, along with appropriate intervention and dietary changes, can lead to an excellent prognosis for individuals with scurvy.

## Introduction

There has been a recent increase in documented cases of scurvy, a condition caused by vitamin C deficiency (1). Vitamin C or ascorbic acid, is an essential micronutrient for the human body. Since humans cannot convert glucose into ascorbic acid, they must rely on dietary sources, mainly fresh fruits and vegetables (2). Ascorbic acid plays a crucial role in the formation of connective tissue, collagen production, and creating dentine and osteoid (3). Due to its involvement in various biological processes, a deficiency in vitamin C can lead to a wide array of clinical symptoms (2). These symptoms often overlap with those of other systemic conditions, such as infectious, hematological, rheumatological, or musculoskeletal disorders (4). Symptoms of vitamin C deficiency usually emerge within one to three months of inadequate intake and can often be identified through a detailed medical and dietary history (5). Scurvy is frequently misdiagnosed because its symptoms resemble those of other diseases, potentially leading to unnecessary and costly tests (6). Diagnosis can be confirmed through laboratory tests or empirical vitamin C supplementation, which typically results in rapid clinical improvement (4). While scurvy is considered rare in Western countries, it still occurs in elderly patients, particularly those with psychiatric disorders, intellectual disabilities, or restrictive diets (7). Additionally, scurvy has re-emerged in high-risk children with mental health conditions, physical disabilities, autism, irregular eating habits, gastrointestinal malabsorption, and chronic kidney diseases (8). This paper discusses the case of a 10-year-old autistic boy who presented with gingival swelling and a limping gait, ultimately diagnosed with scurvy.

## Case presentation

A 10-year-old boy with autism spectrum disorder (ASD) was referred to the Department of Paediatric Dentistry for gingival bleeding and swelling that had been present for the past two weeks. The Paediatric medical team had also examined him due to a limping gait and pain that had been ongoing for the past two months. According to his mother, the patient could no longer participate in sports activities as he had previously done. A comprehensive history was obtained from the mother, who reported that the boy had experienced significant weight loss, having lost four kilograms over the past two months. Additionally, the patient had suffered from multiple oral ulcers for the last two months and had become increasingly lethargic and pale. Over the past month, he appeared progressively cachectic due to a poor appetite. The boy did not experience night sweats, fever, or hair loss and had no other joint pain.

The patient is the third child of four siblings, with the other siblings being healthy. He has controlled bronchial asthma, managed with an MDI Salbutamol inhaler when necessary, and has a history of eczema. There was no history of past surgeries or allergies. At 18 months, the patient was noted to have developmental delay, and he was diagnosed with ASD at the age of three. He is currently under dermatology follow-up for milia on his face. The boy resides in Batu Caves with his parents and a helper. The patient's mother described him as a picky eater. His typical choice of food is limited to rice, potato, carrot, chicken, biscuit and chocolate-flavored beverages (Supplementary Table 1). However, since the onset of oral ulcerations, his oral intake has been significantly reduced. He consumed only small amount of plain soup without vegetables, biscuits and chocolate-flavored beverages (Supplementary Table 2). Regarding dental hygiene, the patient brushes his teeth twice daily using adult toothpaste, with assistance from his mother. While the patient is usually cooperative with brushing and flossing, he has become restless and uncooperative since the onset of gum swelling.

Upon physical examination, the child, accompanied by his mother, was observed to have a thin build. He was not tachypneic, uncooperative, and showed no signs of clubbing. He was also limping as he entered the clinic. Extra-orally, no significant findings were noted, with no facial asymmetry, limited mouth opening, or palpable lymph nodes. His weight was recorded as 25 kg (10th percentile) and his height as 135 cm (25th percentile). Intra-orally, the patient had fair oral hygiene and halitosis, with mixed dentition. Localized areas of erythematous and hypertrophic gingiva were observed (Supplementary Figure 1). The gums appeared red and purplish, and they bled easily upon contact. The swelling was present in all four quadrants of the mouth.

A series of investigations were conducted by paediatric medical team prior to the referral, including venous blood gas analysis, erythrocyte sedimentation rate (ESR), full blood count, renal profile, liver function tests, complement blood tests for component 3 (C3) and component 4 (C4), C-reactive protein levels (CRP), International normalized ratio (INR), serum muscle enzyme tests, coagulation tests, and ferritin levels. All these results were within normal ranges. A peripheral blood film, full blood count and ferritin analysis showed unremarkable findings, as outlined in Supplementary Tables 3, 4. The Mycoplasma serology test also returned negative. However, the patient's Interleukin-6 level (IL-6) was slightly elevated at 13.04 pg/ml, with a normal range of <6 pg/ml, indicating the presence of inflammation.

The patient was observed to have difficulty squatting and sitting on a bench, requiring support by holding onto a rail or placing his hands on his knee. He also had trouble standing from a sitting position. On physical examination, there was no tenderness over the left hip, although the patient was unable to pinpoint a specific tender area. The range of motion in both the hip and knee joints was full, with no swelling, redness, or increased warmth noted. Plain radiographs of the patient's hip, bilateral knees, and femur were taken and showed no signs of rickets, no increased joint space, and no fractures (Supplementary Figure 2). They also observed multiple perifollicular hemorrhages on both of his limbs, as well as tenderness on his right calf. However, it was not warm to the touch.

Based on our impression of scurvy, the paediatric medical team has conducted more investigations to support the diagnosis. Multiple visible corkscrew hairs were noted on his bilateral lower limbs under magnification, which were observed using lignocaine gel and an ophthalmology tool. The patient's blood sample was sent to Australia for vitamin C plasma level testing, with results available after almost a month. The test revealed a significantly low level of vitamin C (5 µmol/L), well below the normal range of 28–120 µmol/L (Supplementary Table 5).

The patient's response to vitamin C supplementation was remarkable. Within seven days, there was a notable improvement in his general health and gingival appearance (Supplementary Figure 3).

During the one-week follow-up, the patient's limping gait had improved, and his gums no longer bled easily upon contact. Complete healing of the gingiva was observed within 16 days of starting vitamin C supplementation, and his weight increased from 25 kg to 26 kg. The patient's mother reported no bleeding while brushing or flossing his teeth. The limping gait had significantly improved, and there were no oral ulcers for the past week. After a month and a half, the patient's initial symptoms were almost completely resolved. His mother noted that he no longer limped, had regained his appetite, was more cheerful, and could run and jump. Five months from the initial visit, the patient weighed 30 kg.

## Discussion

Although scurvy was long believed to be a disease of the past, recent reports indicate its re-emergence, particularly in specific groups. Children with developmental delays, cerebral palsy, autism, oral aversions, and those with diets high in dairy but lacking fruits and vegetables are among those most affected (9). However, scurvy can also impact otherwise healthy individuals, especially in children who are often labeled as “picky eaters” or those with highly selective diets, which typically exclude fruits and vegetables, leading to vitamin C (ascorbic acid) deficiency (10).

Ascorbic acid plays a crucial role in collagen synthesis, which is why early manifestations of its deficiency primarily affect the gums, skin, and bones (10). Diagnosing scurvy remains challenging because it is considered rare, and there is a misconception that nutritional deficiencies only occur in underdeveloped countries. Therefore, physicians need to obtain a comprehensive dietary history to make an accurate diagnosis (11).

Autism Spectrum Disorder (ASD) is a developmental disorder characterized by limited communication, a lack of social interaction, and restricted interests. Studies show that 46%–89% of individuals with ASD have restrictive diets, often consisting primarily of carbohydrates, snacks, and processed foods, with little to no intake of fruits and vegetables (7). This report discusses a case of scurvy in a 10-year-old boy with ASD, who exhibited selective eating behaviors and completely excluded foods rich in ascorbic acid. As he grew older, his food preferences became even more selective, and he refused most of fruits and vegetables. The recent incident of multiple oral ulcers since two months ago made his food aversion worsen with total exclusion of fruit and vegetables that could have triggering the onset. It typically takes only one to three months of vitamin C deficiency for the initial signs of scurvy to appear (12).

The main clinical presentations of scurvy include gingival swelling and bleeding, arthralgia, limb pain, irritability, pseudoparalysis, and nutritional issues (13). Around 80% of patients with scurvy experience musculoskeletal symptoms, which are typically later signs and include muscle hypotrophy, swelling, myalgia, and joint pain. The lower limbs, especially the knees, are most commonly affected (7). In our case, the patient's mother first noticed his limping gait, followed by gradual weight loss. He also stopped participating in daily walks and sports activities due to limb pain. Initially, the patient was suspected to have Multisystem Inflammatory Syndrome (MIS-C), prompting several blood tests to rule out other conditions.

Although the clinical manifestations of vitamin C deficiency can overlap with other systemic diseases, such as hematological conditions, infections, or rheumatological disorders, scurvy was suspected in this patient due to his unremarkable test results, selective eating habits, and oral signs such as gum inflammation and limping gait (7). Another key diagnostic clue was the presence of multiple perifollicular hemorrhages and corkscrew hairs on his bilateral lower limbs, observed under magnification. According to Maxfield et al., classic signs of vitamin C deficiency include perifollicular hemorrhages, corkscrew hairs, gingival bleeding, anorexia, and fatigue (14). While scurvy can still be misdiagnosed due to its broad clinical spectrum, when correctly identified, it has an excellent prognosis with vitamin C supplementation. Common misdiagnoses include ulcerative gingivitis, vasculitis, hematological abnormalities, blood dyscrasias, infections, trauma, or medication side effects (15). This case highlights the importance of obtaining a detailed and thorough history, especially for pediatric patients or those at risk (16).

For this patient, a blood test was sent to Australia to measure serum vitamin C levels after scurvy was suspected. The results were expected in two to three weeks, so the patient was started on vitamin C as both a diagnostic and therapeutic measure while awaiting the results, as vitamin C is safe and easy to administer. A study by Monroig-Rivera et al. recommends empiric oral vitamin C in cases of suspected vitamin C deficiency, especially when patients present with musculoskeletal complaints of unknown origin. This approach helps to rule out other conditions such as infections, inflammations, or trauma-related swelling and muscle weakness, while waiting for further diagnostic results (17).

There is no universally agreed-upon dosage for vitamin C in the treatment of scurvy. However, the main goal is to restore vitamin C levels in the body to alleviate symptoms. One study found that administering 100 mg of vitamin C three times daily led to improvement in symptoms by the first day, with skin lesions resolving within weeks (18). Another study suggested a regimen of 500 mg of vitamin C twice daily, which resulted in rapid pain relief within four days and a significant improvement in overall condition (6). Two other case reports, including one involving a child, showed that administering 500 mg of vitamin C daily led to quick improvement in both pain and general health within days (1). Additionally, a case involving a 3-year-old child with picky eating habits saw significant improvement in strength after just one dose of 100 mg of vitamin C three times daily, and the child was discharged shortly thereafter (19).

Nevertheless, for patients with gastrointestinal issues or compromised nutrient absorption, oral vitamin C supplementation may not be effective. In such cases, intravenous (IV) vitamin C is recommended to raise and stabilize serum vitamin C levels to within the normal range (20).

In this case, we decided to start the patient on 200 mg of Vitamin C three times a day for a month, followed by a reduction to 200 mg twice a day and eventually 200 mg once daily. Additionally, the patient's parents were advised to include vitamin C-rich foods in his diet to further support his recovery. Within the first week of supplementation, significant improvements were observed in both the patient's oral health and his ability to walk without limping. The patient was also reported to be less anxious, and his body weight began to increase. At the one-and-a-half-month review, the patient had returned to his cheerful self, and all of his initial symptoms had completely resolved. He was able to run and play as he normally would, indicating a full recovery.

## Conclusion

In conclusion, this case highlights the occurrence of scurvy in a 10-year-old boy with autism and selective eating habits. As demonstrated in this case, food preferences and limited dietary choices in children with autism spectrum disorder (ASD) can contribute to nutritional deficiencies. Early diagnosis of scurvy, along with a multidisciplinary approach involving dietitians, behavioral therapists, and caregivers, can significantly impact the overall health and developmental progress of these children. This case serves as a reminder that patients at high risk for nutritional deficiencies require careful attention to their specific dietary preferences and needs. While scurvy may seem like a disease of the past, when diagnosed early, it is a treatable condition. The key takeaway is that timely intervention can lead to remarkable improvements in both physical health and well-being.

## Parents’ perspective

As a parent, I was aware of scurvy but never imagined it could affect my child. When my child first exhibited symptoms such as a limp, loss of appetite, and weight loss, we were worried but uncertain about the cause. The initial diagnosis from the Paediatric Medical team was Multi-System Inflammatory Syndrome in Children (MIS-C), which seemed plausible then. With no family history of similar conditions, we followed the recommended course of action. However, when my child also developed gum swelling, we were referred to the dental team for further assessment.

Within a week, his condition deteriorated—he became increasingly lethargic, struggled to climb out of the swimming pool, experienced severe calf pain, and his gum swelling worsened, accompanied by bleeding. At this stage, we took him to the Paediatric Dentistry department, where he was immediately diagnosed with scurvy based on his clinical symptoms. Treatment with a Vitamin C supplement was well tolerated, and within a few days, he showed significant improvement.

To other parents, I encourage you to trust your instincts. If something doesn’t seem right, don’t hesitate to seek medical advice. Your observations can be crucial in reaching an accurate diagnosis. I am incredibly grateful to the Paediatric Dentistry team for their timely and precise diagnosis and prompt intervention, which was instrumental in my child's recovery.

## Data Availability

The raw data supporting the conclusions of this article will be made available by the authors, without undue reservation.
