# Supplementary material for: Case Report: Autistic child with restrictive eating behaviour, limping gait and erythematous gingival mass-scurvy?
Source: Front Child Adolesc Psychiatry. 2025 Jul 18;4:1600861. doi: 10.3389/frcha.2025.1600861 (PMC12313698; doi:10.3389/frcha.2025.1600861)
Supplement: Supplementary file 1 [file Table1.docx]

Supplementary Material

# Supplementary Data

Nil

# Supplementary Figures and Tables

## Supplementary Figures


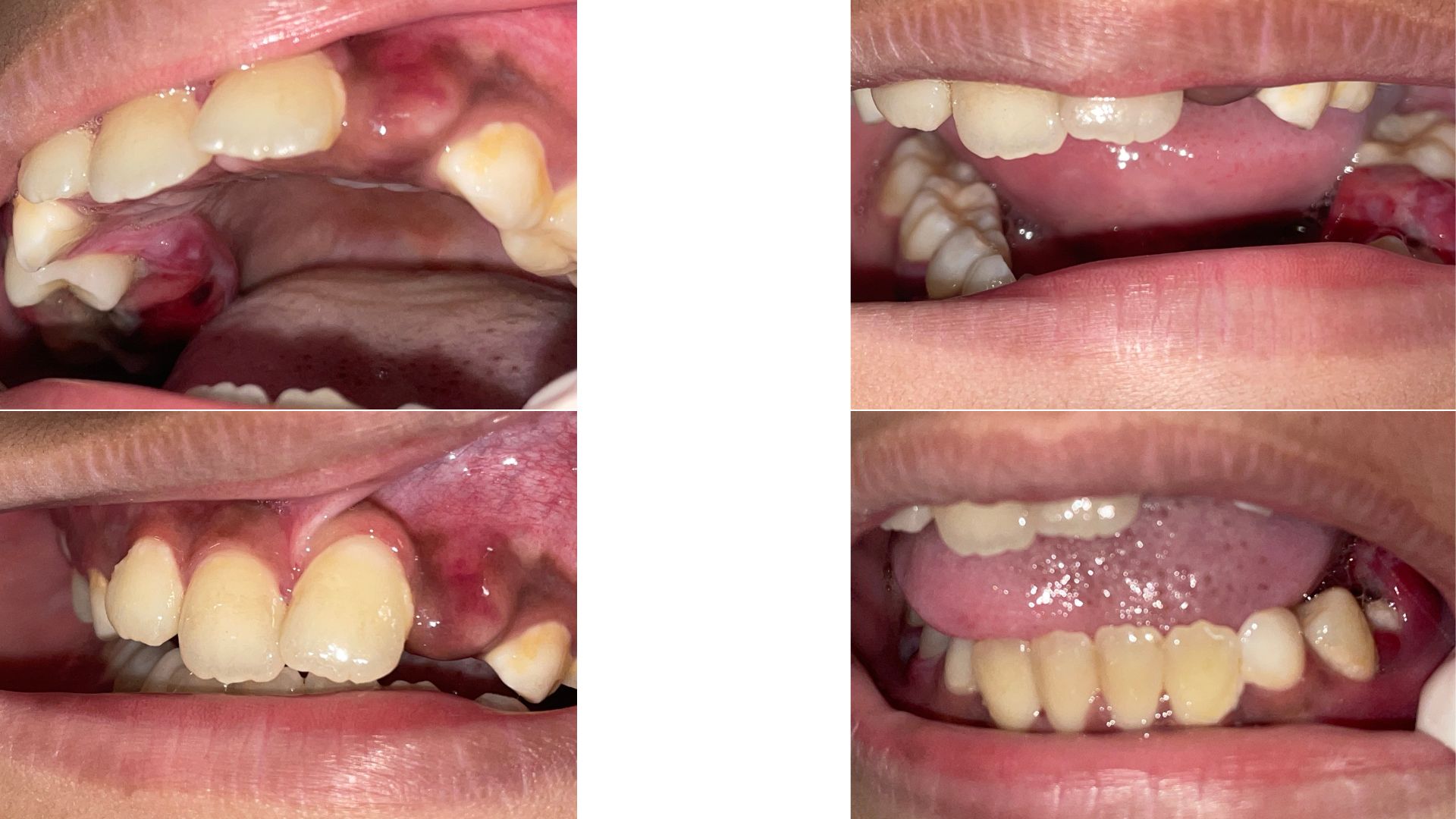

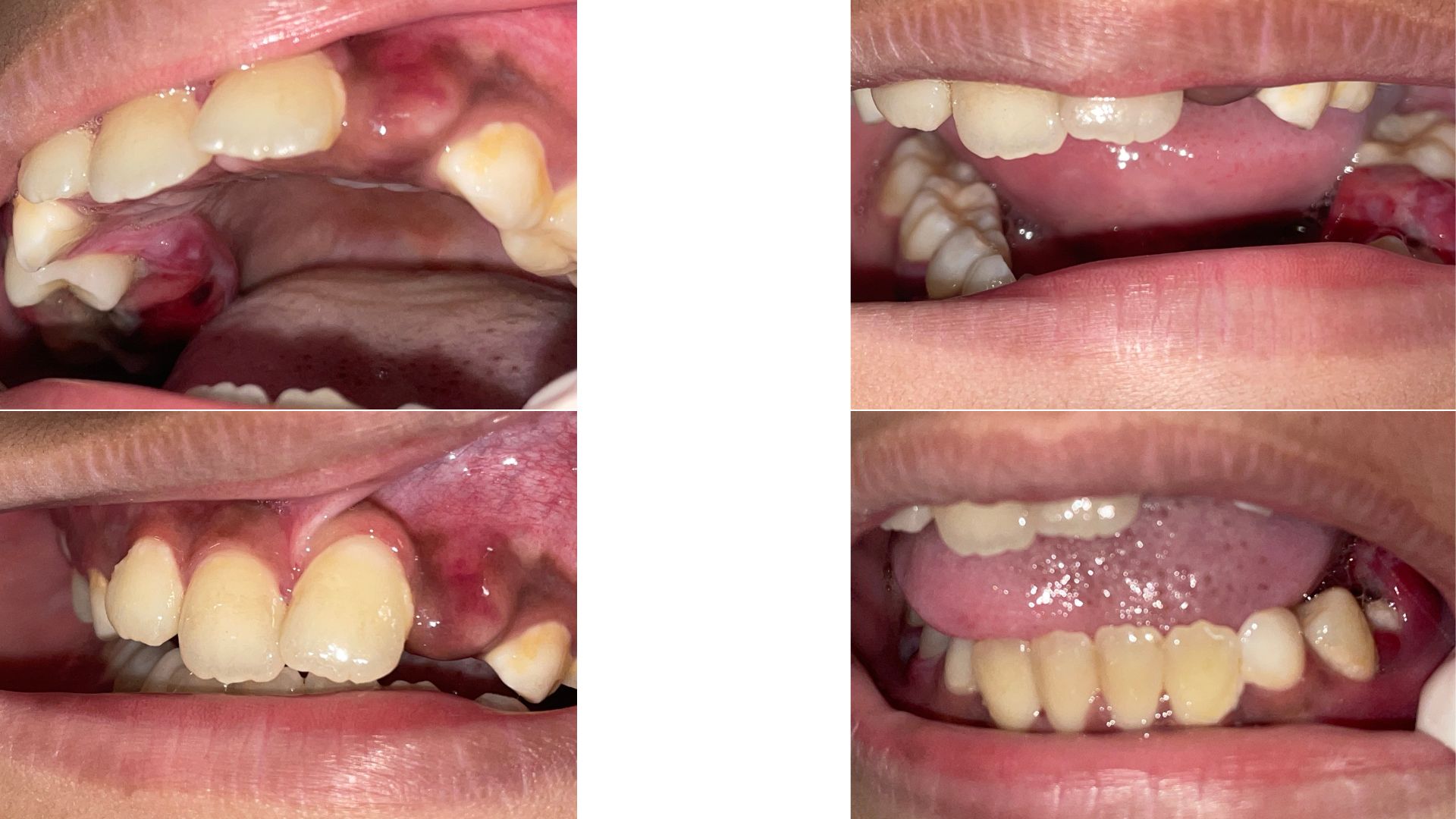

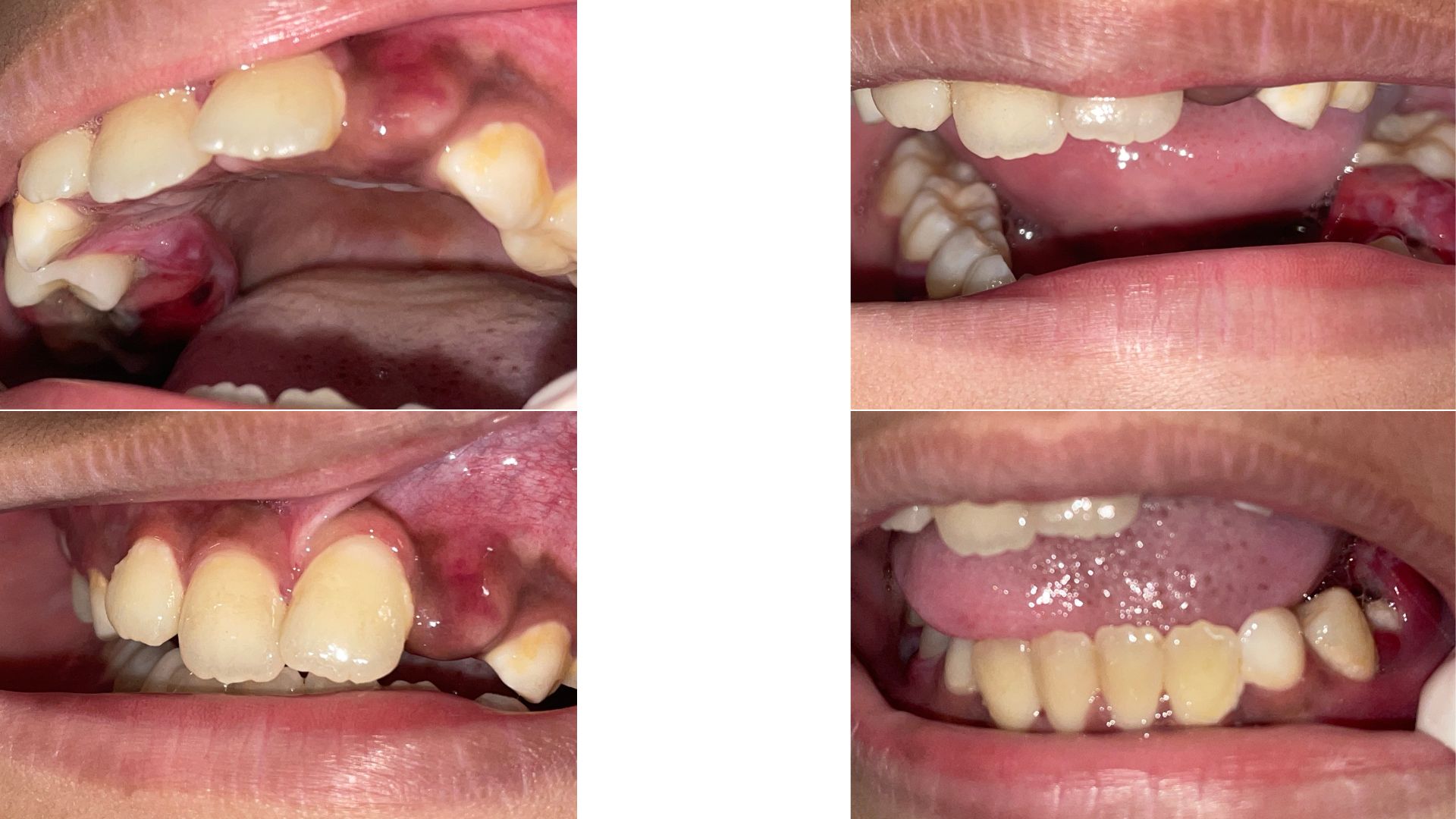

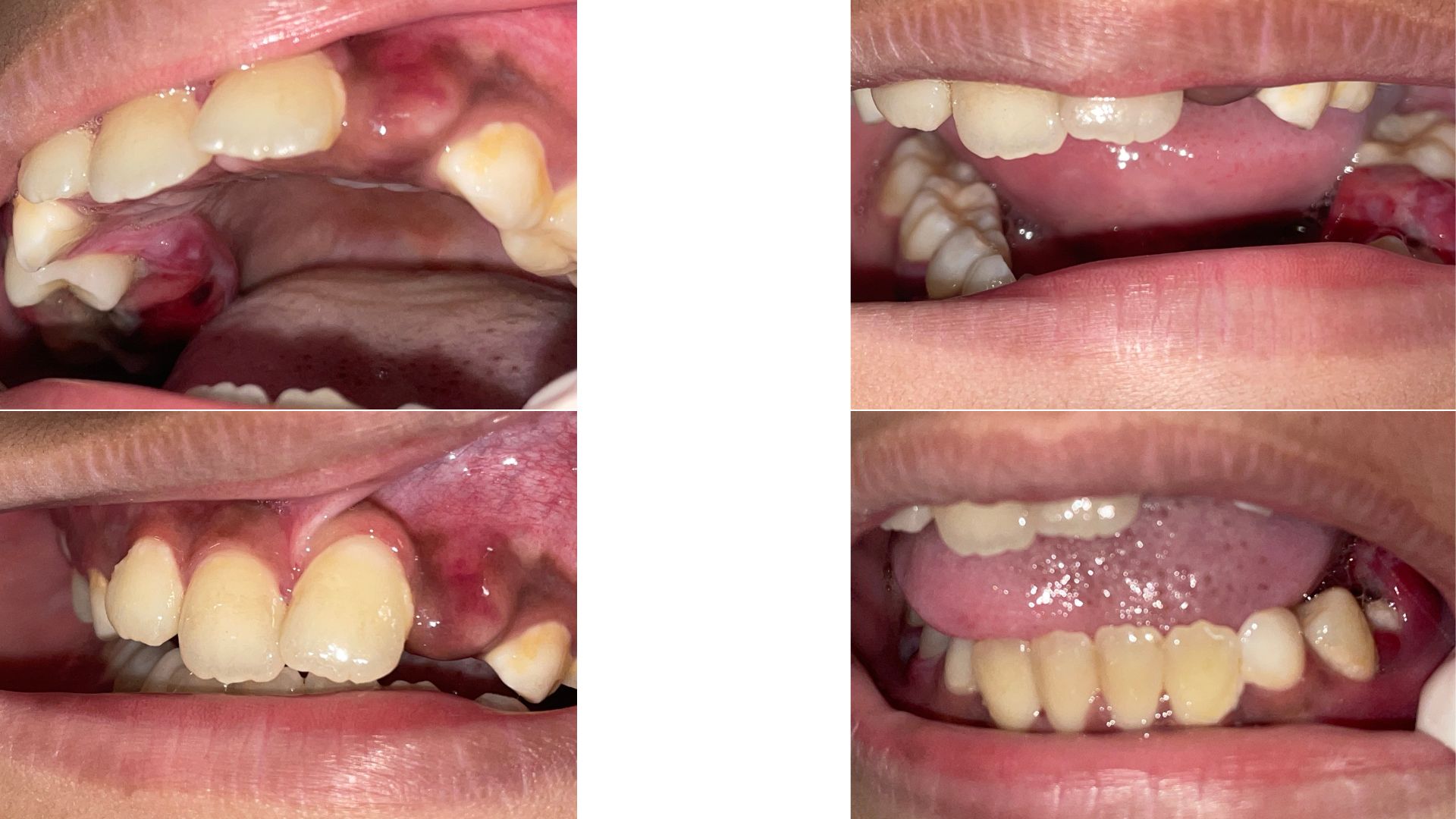


D

C

B

A

**Supplementary Figure 1.** A) Reddish-purple mass of gingiva at tooth 55 and 14 palatal region (red circle). (B) Erythematous tissue overlying unerupted tooth 22 area with visible and palpable bulge (blue circle). (C) Reddish-purple mass of gingiva at tooth 34 and 75 occlusal region (blue arrow). (D) Reddish-purple gingival swelling at interdental of tooth 44 and 85 buccal region (red arrow).


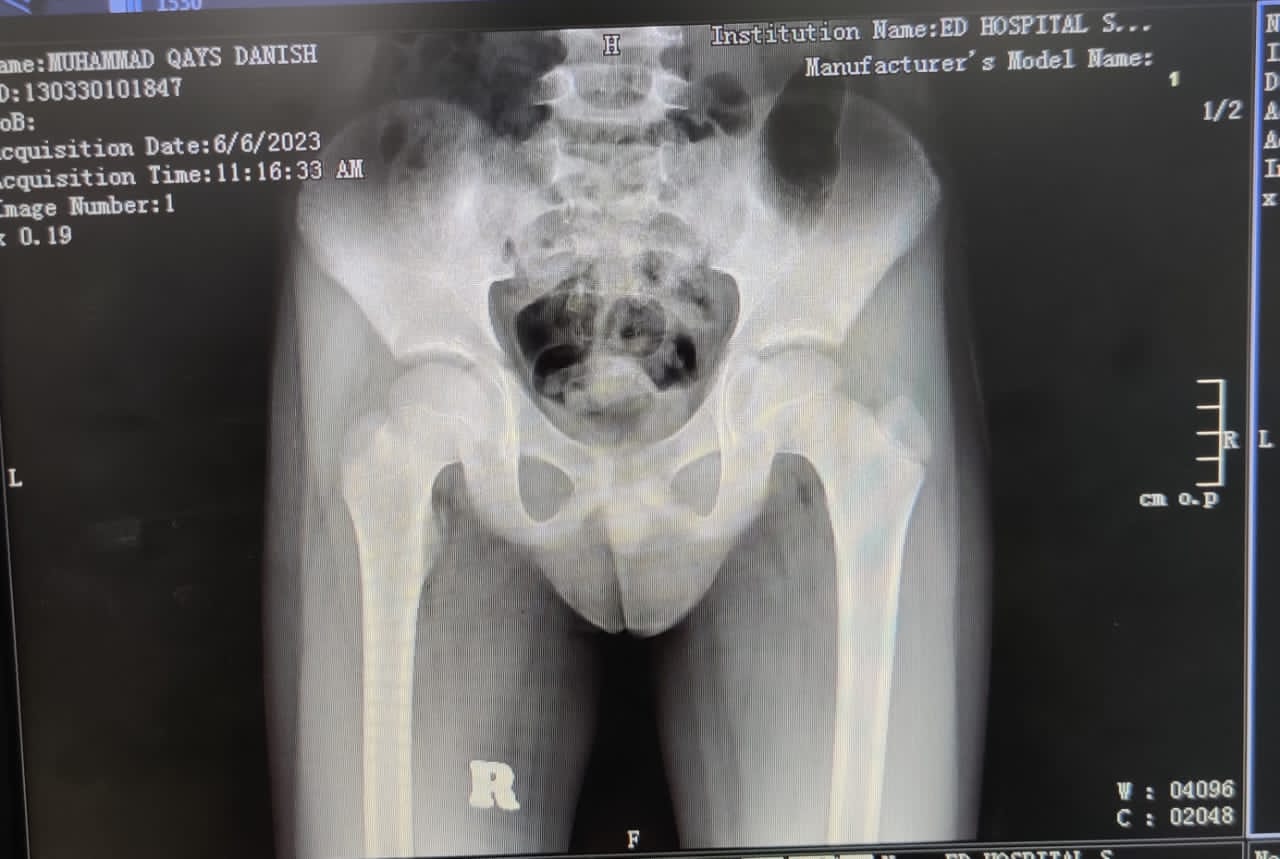

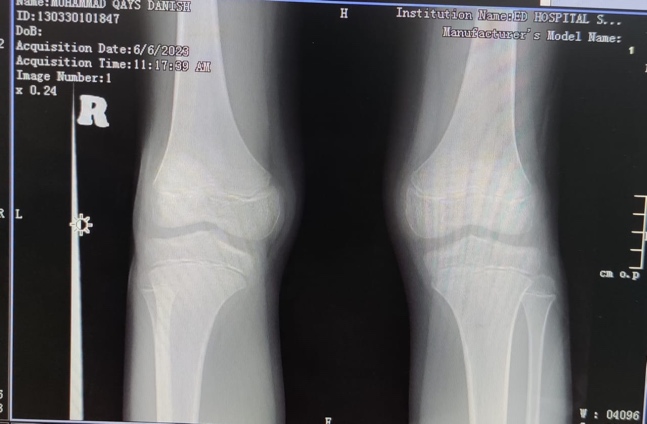


B

A


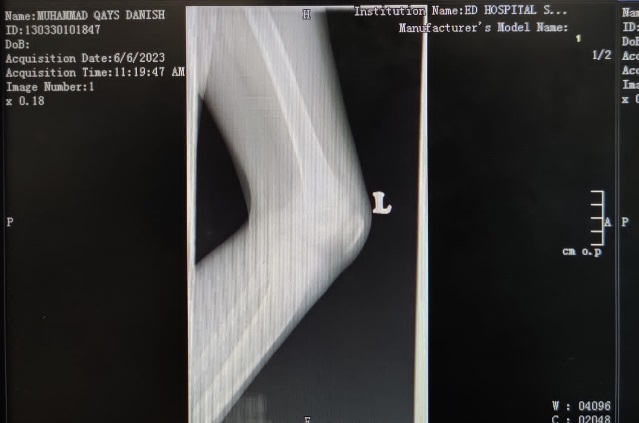

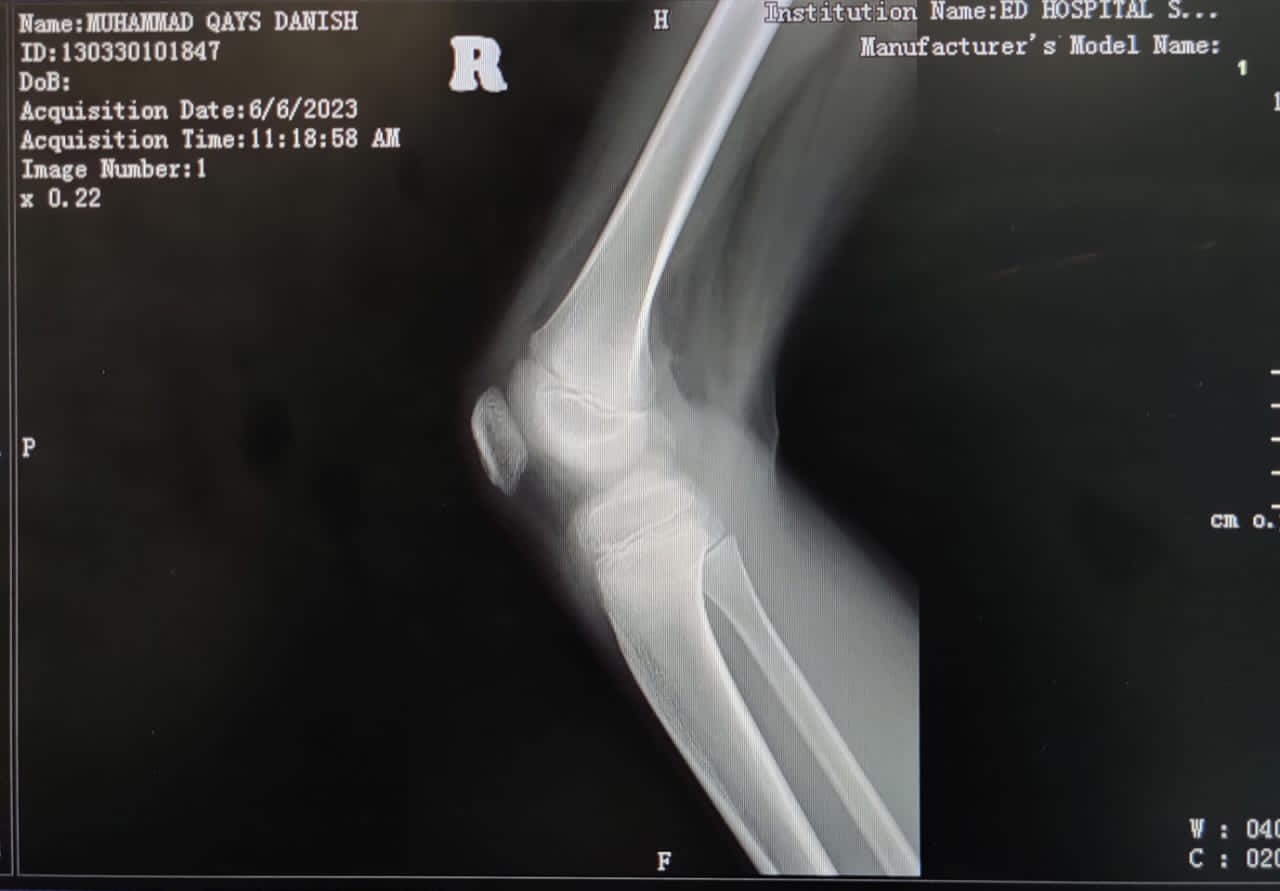
**Supplementary Figure 2.** Pain radiograph of patient's; (A) Hip (B) Bilateral knee (C) Left knee (D) Right knee

D

C


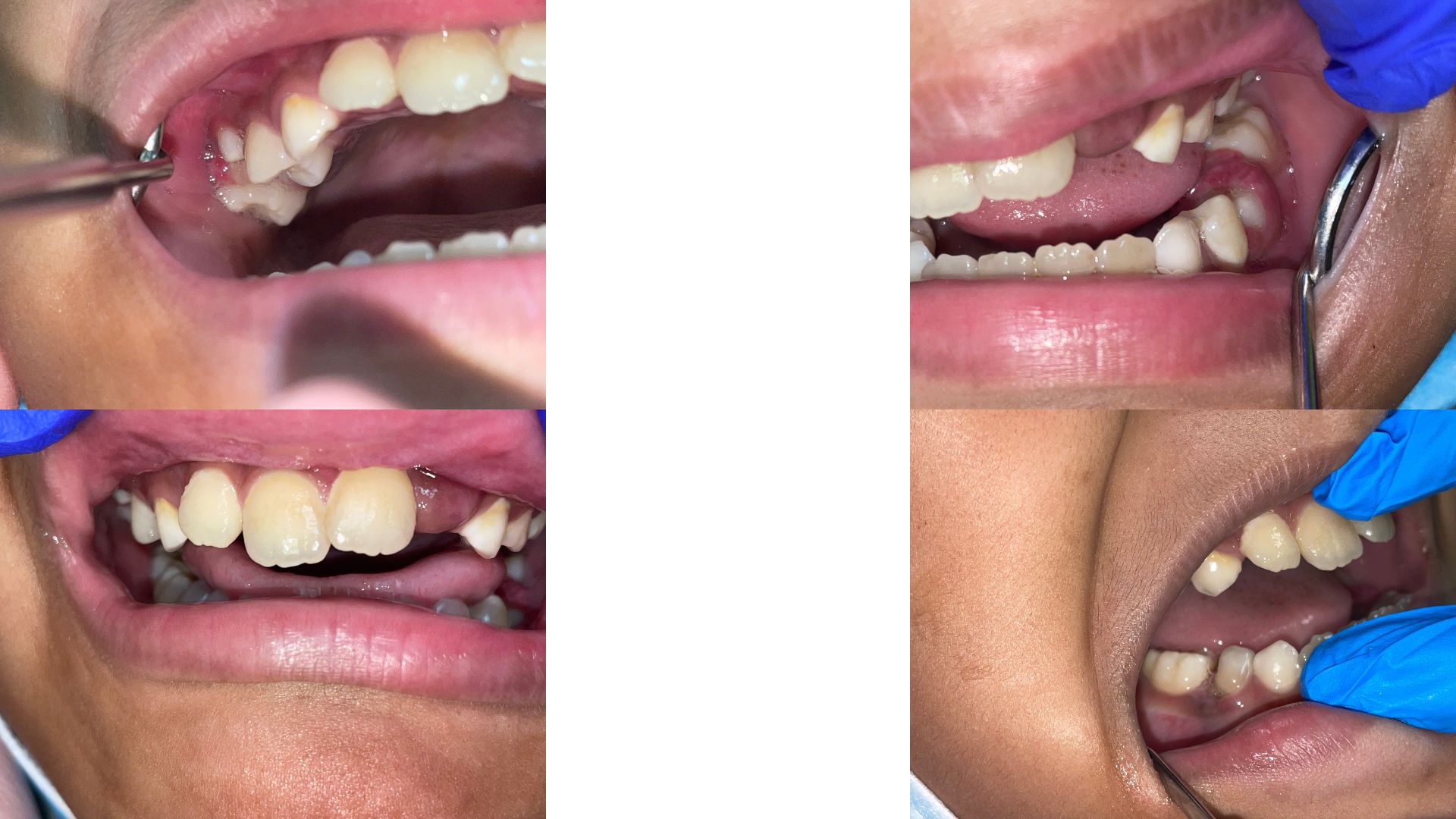

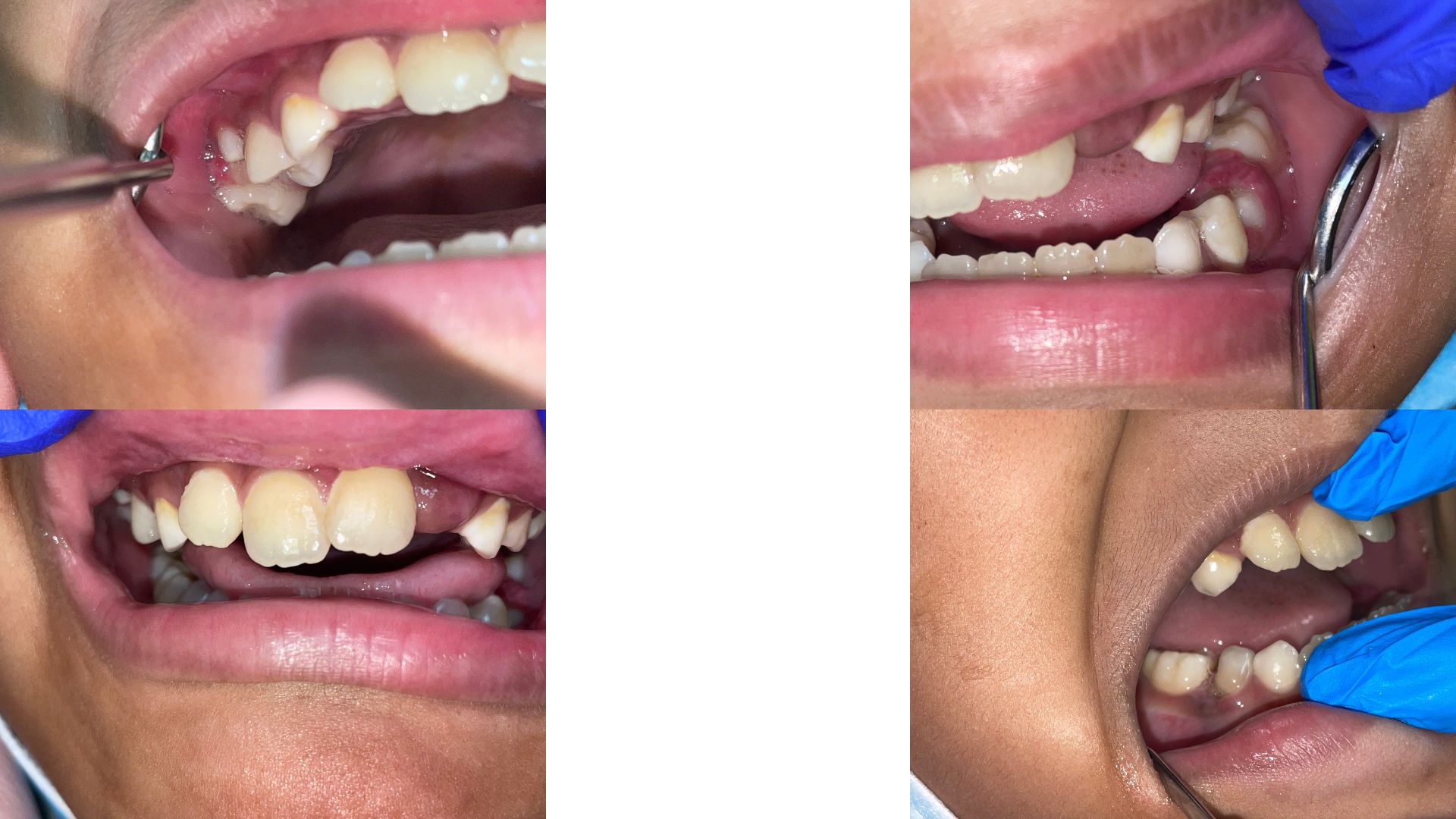

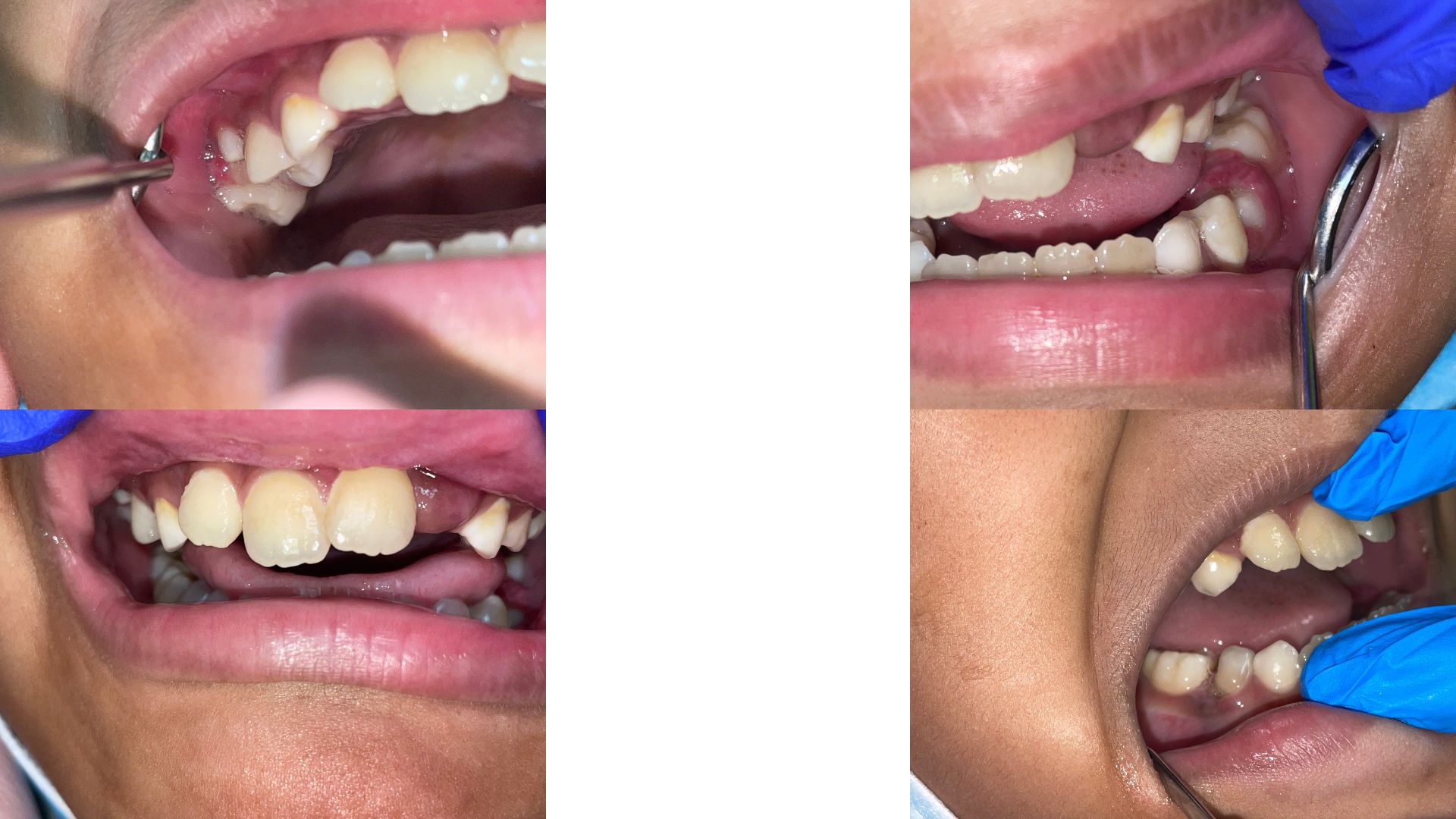

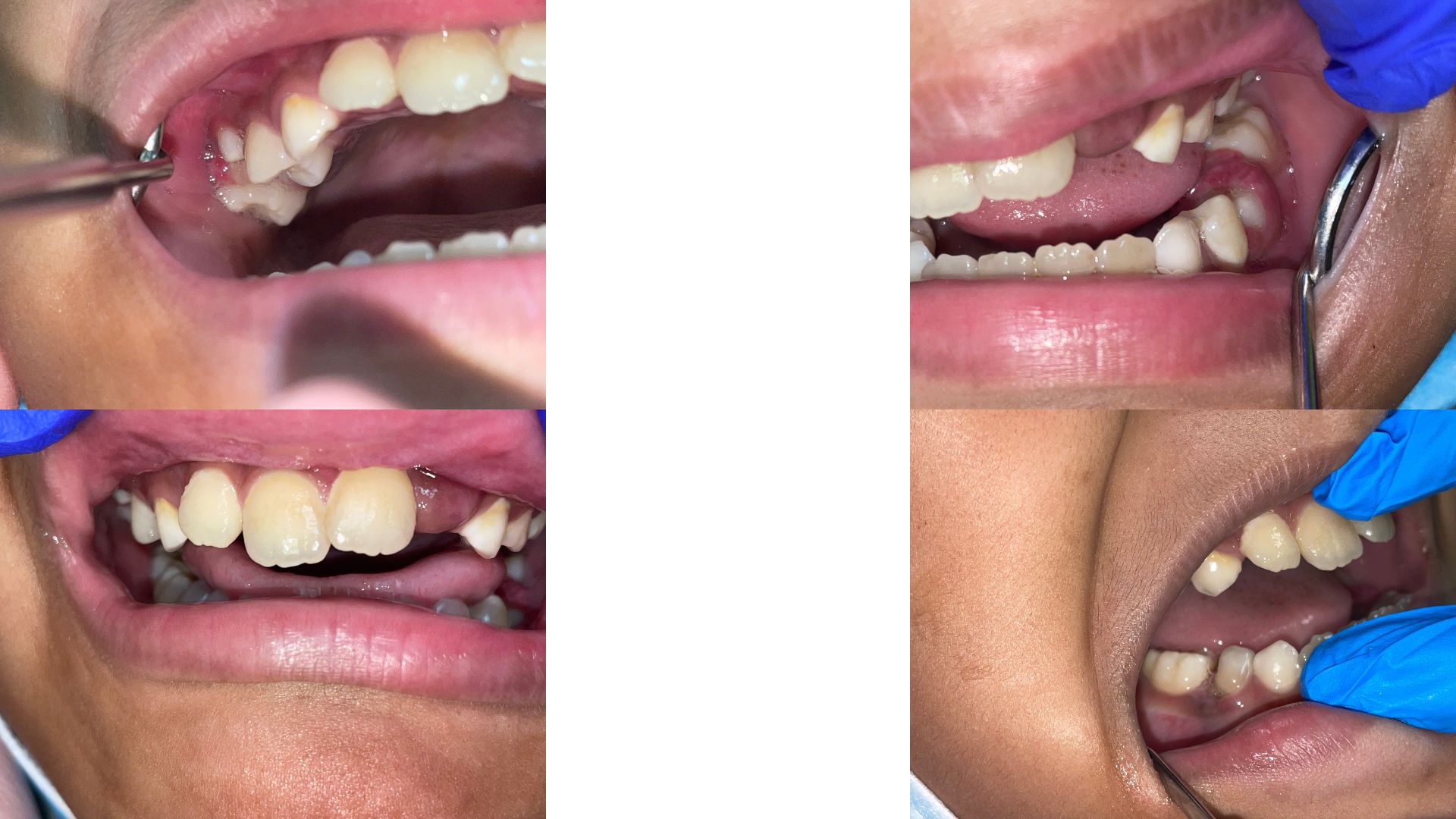


**Supplementary Figure 3.** A) Pinkish mass of gingiva at tooth 14 and 55 palatal region decreased in size (red circle). (B) Pinkish tissue overlying unerupted tooth 22 area with visible and palpable bulge (blue circle). (C) Pinkish mass of gingiva at tooth 34 and 75 occlusal region decreased in size (blue arrow). (D) Resolved gingival swelling at interdental of tooth 44and 85 buccal region (red arrow).

**Table 1. Dietary diary of patient’s routine daily meal**

| **Meal time** | **Type of diet** | **Portion** |
| --- | --- | --- |
| Breakfast | Tea drink | 1 cup |
| Lunch | Rice  Chicken and carrot soup | ½ bowl  1 small piece chicken and 3 small pieces carrot |
| Tea time | Tea/chocolate drink  Biscuits | 1 cup  2 pieces |
| Dinner | Rice  Chicken and carrot soup | ½ bowl  1 small piece chicken and 3 small pieces carrot |

**Table 2. Dietary diary two months before the onset of Scurvy**

| **Meal time** | **Type of diet** | **Portion** |
| --- | --- | --- |
| Breakfast | Biscuit | 2 pieces |
| Lunch | Plain soup | ½ bowl |
| Dinner | Chocolate drink | ½ cup |

**Table 3. Peripheral blood film laboratory results**

| **Blood components** | **Peripheral blood film** |
| --- | --- |
| Hemoglobin | Within normal range. Rectic count 1.34% |
| Red blood cell | Normochromic normocytic RBC seen |
| White blood cell | Within normal range. No blast cell seen |
| Platelet | Adequate |

**Table 4. Full blood count and Ferritin laboratory results**

| **Blood components** | **Value** | **Ref. Range** |
| --- | --- | --- |
| Hemoglobin | 13.6 g/dL | (13.5 - 17.4) |
| White blood cell | 6.28 x 10^9^/ L | (4.08 - 11.37) |
| Platelet | 284 x 10^9^/ L | (142 - 350) |
| Mean Corpuscular Volume | 86 fL | (80.6 - 95.5) |
| Mean Corpuscular Haemoglobin | 27 pg | (26.9 - 32.3) |
| Ferritin | 111.6 ng/mL | Notes:  IRON DEFICIENCY  Children >= 5 years old : <15  In inflammation, Iron deficiency is defined as;  Children >= 5 years old : <70 |

**Table 5. Plasma Vitamin C levels**

| **Specimen** | **Ascorbic Acid** | **Unit** | **Ref. Range** |
| --- | --- | --- | --- |
| Plasma | 5 | µmol/L | (28-120) |
